# Increasing incidence of *Plasmodium ovale* and persistent reporting of *Plasmodium vivax* in imported malaria cases: an analysis of 9-year surveillance data in four areas of China

**DOI:** 10.3389/fpubh.2023.1203095

**Published:** 2023-06-28

**Authors:** Xiaoxiao Wang, Wenjie Xu, Fei Luo, Kangming Lin, Tao Zhang, Linong Yao, Xuan Zhang, Jiaqi Zhang, Sarah Auburn, Duoquan Wang, Wei Ruan

**Affiliations:** ^1^Department of Infectious Diseases, Zhejiang Center of Disease Control and Prevention, Hangzhou, China; ^2^Department of Endemic and Parasitic Diseases, Chongqing Center for Disease Control and Prevention, Chongqing, China; ^3^Department of Infectious Diseases, Guangxi Center of Disease Control and Prevention, Nanning, China; ^4^Department of Infectious Diseases, Anhui Center of Disease Control and Prevention, Hefei, China; ^5^Global and Tropical Health Division, Menzies School of Health Research and Charles Darwin University, Darwin, NT, Australia; ^6^Nuffield Department of Medicine, Centre for Tropical Medicine and Global Health, University of Oxford, Oxford, United Kingdom; ^7^National Institute of Parasitic Diseases, Chinese Center for Disease Control and Prevention, Shanghai, China; ^8^School of Global Health, Chinese Center for Tropical Diseases Research, Shanghai Jiao Tong University School of Medicine, Shanghai, China

**Keywords:** imported malaria, China, Western Africa, migrant workers, surveillance, medical visit pattern

## Abstract

**Background:**

This study aimed at exploring the epidemiological pattern of imported malaria in China before malaria elimination in 2021, to provide evidence-based data for preventing malaria re-establishment in China.

**Methods:**

Nine-year surveillance data on imported malaria in four provincial-level administrative divisions (PLADs) (Anhui, Chongqing, Guangxi, and Zhejiang) between 2011 and 2019 were thoroughly collected and analyzed.

**Results:**

A quite stable trend in imported malaria cases between 2011 and 2019 was observed. In total, 6,064 imported patients were included. *Plasmodium falciparum* was the most frequently reported species (4,575, 75.6%). Cases of malaria were most frequently imported from Western Africa (54.4%). We identified an increasing trend in *P. ovale* and a persistence of *P. vivax* infections among the cases of malaria imported from Western Africa. Most patients (97.5%) were 20–50 years old. Among imported malaria infections, the main purposes for traveling abroad were labor export (4,914/6,064, 81.0%) and business trips (649, 10.7%). Most patients (2,008/6,064, 33.1%) first visited county-level medical institutions when they sought medical help in China. More patients were diagnosed within 3 days after visiting Centers for Disease Control and Prevention (CDCs) or entry–exit quarantine facilities (EQFs) (1,147/1609, 71.3%) than after visiting medical institutions (2,182/3993, 54.6%).

**Conclusion:**

Imported malaria still poses a threat to the malaria-free status of China. County-level institutions are the primary targets in China to improve the sensitivity of the surveillance system and prevent the re-establishment of malaria. Health education should focus on exported labors, especially to Western and Central Africa. Increasing trend in *P. ovale* and persistence of *P. vivax* infections indicated their underestimations in Western Africa. Efficient diagnostic tools and sensitive monitoring systems are required to identify *Plasmodium* species in Africa.

## 1. Introduction

Malaria is a parasitic disease with a long history in humans and is common in tropical and subtropical regions. According to the World Malaria Report (1), the disease affected an estimated 241 million people and caused 627,000 deaths in 2020. Of all these cases of malaria, most occurred in the World Health Organization (WHO) African Region, which accounted for 95% of reported cases, followed by the WHO Eastern Mediterranean Region (2.3%) and the WHO South-East Asian Region (2%). In response to the great efforts of the WHO Global Malaria Program and its partners, the malaria burden has decreased substantially over the past decade. Between 2010 and 2020, the total number of cases of malaria in the 21 countries that participated in the WHO “eliminating countries for 2020” (E-2020) initiative was reduced by 84% (2). Cases of and deaths from malaria have also fallen sharply in other regions.

China was one of the countries involved in E-2020 (2). According to the criteria of the Global Technical Strategy for Malaria 2016–2030 and under a country-led and country-owned endeavor, China has been certified a malaria-free country in 2021, after the implementation of an integrated strategy for malaria control, coupled with the country’s socioeconomic and environmental development. Since 2017, all cases of malaria reported in China have been imported malaria, and imported malaria poses a non-negligible threat to China’s malaria-free status. Most of these cases came from the African Region, with some from South-East Asia. Under the Belt and Road Initiative, and with the increasing globalization, labor exportation, and business cooperation, the reintroduction of local malaria transmission in currently malaria-free areas is highly possible. Besides continued surveillance of the distribution and insecticide resistance of malarial vector mosquitoes (3), the rapid identification of imported malaria and its timely treatment before the parasite is transmitted to mosquitoes are also essential in preventing the re-establishment of local transmission (4).

During the period in which malaria was eliminated, China instituted a nationwide malaria-specific surveillance system, and cases of malaria were reported in a real-time, web-based manner (5). A network of national reference laboratories was also created, facilitating case detection, with the blind assessment of samples. Local annual team-based microscopy competitions also maintained a capacity for the rapid and accurate diagnosis of malaria. However, as the number of imported cases of malaria has gradually increased, China’s malaria-free status is potentially threatened. To understand the epidemiological characteristics of the imported malarial species, and the timeliness of their diagnosis and treatment, we retrospectively analyzed malaria surveillance data on cases of imported malaria in four provincial-level administrative divisions (PLADs) in China (Anhui province, Chongqing Municipality, Guangxi Zhuang Autonomous Region, and Zhejiang Province) reported between 2011 and 2019, which have relatively high numbers of imported malaria cases. This information should allow the development of further preventive and control strategies to avert the potential reintroduction of locally transmitted malaria and of more sophisticated health education.

## 2. Methods

### 2.1. Data source

Data on the malarial infections in the study area were obtained from the Information System for Parasitic Disease Control and Prevention (ISPDCP) (Supplementary Figure S1). The basic information collected for further analysation including sex, age, occupation, residence, and details of the malarial illness, such as the date of symptom onset, the date of diagnosis, and the date of treatment. After a patient was first diagnosed with malaria, the case should be reported into the ISPDCP within 24 h. Briefly, the county-level CDCs are responsible for case confirmation and investigation, to collect the relevant details of the patient, including travel history, dates of departure from and arrival back to China, countries visited, and purpose of travel. Laboratory reviews are preliminarily done by CDCs at county (sometimes municipal) level using microscopy method and/or rapid diagnostic test (RDT), and finally reviewed by provincial CDCs, using microscopy, RDT and PCR techniques. All the epidemiological information and malaria species will be submit to the ISPDCP, and also included in our analysis.

A total of 6,126 cases of imported malaria in the four PLADs studied were downloaded from the ISPDCP. Sixty-two were excluded because of incomplete information, and 6,064 cases were finally included.

### 2.2. Case definition

The case definition of malaria in China was based on a national guideline. The diagnosis was made based on the clinical symptoms, travel history, microscopy test, rapid diagnostic test and PCR. An imported malaria case was defined as “a malaria infection traced to an origin in a malaria-endemic area outside China and within 1 month after returning from the endemic area,” according to the surveillance scheme of the National Malaria Elimination Program. Imported malaria cases would be laboratory-diagnosed, have a travel history to malaria-endemic areas outside of China during the local malaria transmission season, and the disease onset occurred < 1 month after returning to China during the local transmission season.

### 2.3. Data analysis

Statistical analyses were performed with R Project version 3.2.5^1^ and Microsoft Office Excel 2019 (Los Angeles, CA, United States). Two different reviewers pooled the data from the four PLADs studied and reviewed the database independently, deleting cases with missing data on important indicators. The data are described with descriptive statistics. Bar plots and pie plots were used to visualize the numbers and proportions of malaria cases. Box and whisker plots were used to describe the temporal pattern of imported malaria cases. We used kernel density estimation to create smooth curves with which to assess the temporal changes in the numbers of malaria cases over time. Simple linear regression was used to test for linear temporal trends. A difference was considered significant at *p* < 0.05. All reported *p* values are two-tailed.

## 3. Results

### 3.1. General description

Among the 6,064 cases of imported malaria detected in 2011–2019, 1,088 (17.9%) were reported in Anhui, 267 (4.4%) in Chongqing, 3,107 (51.3%) in Guangxi, and 1,602 (26.4%) in Zhejiang. Only 10 cases were in non-Chinese subjects.

### 3.2. Imported malaria trends and seasonality

**Figure 1:**
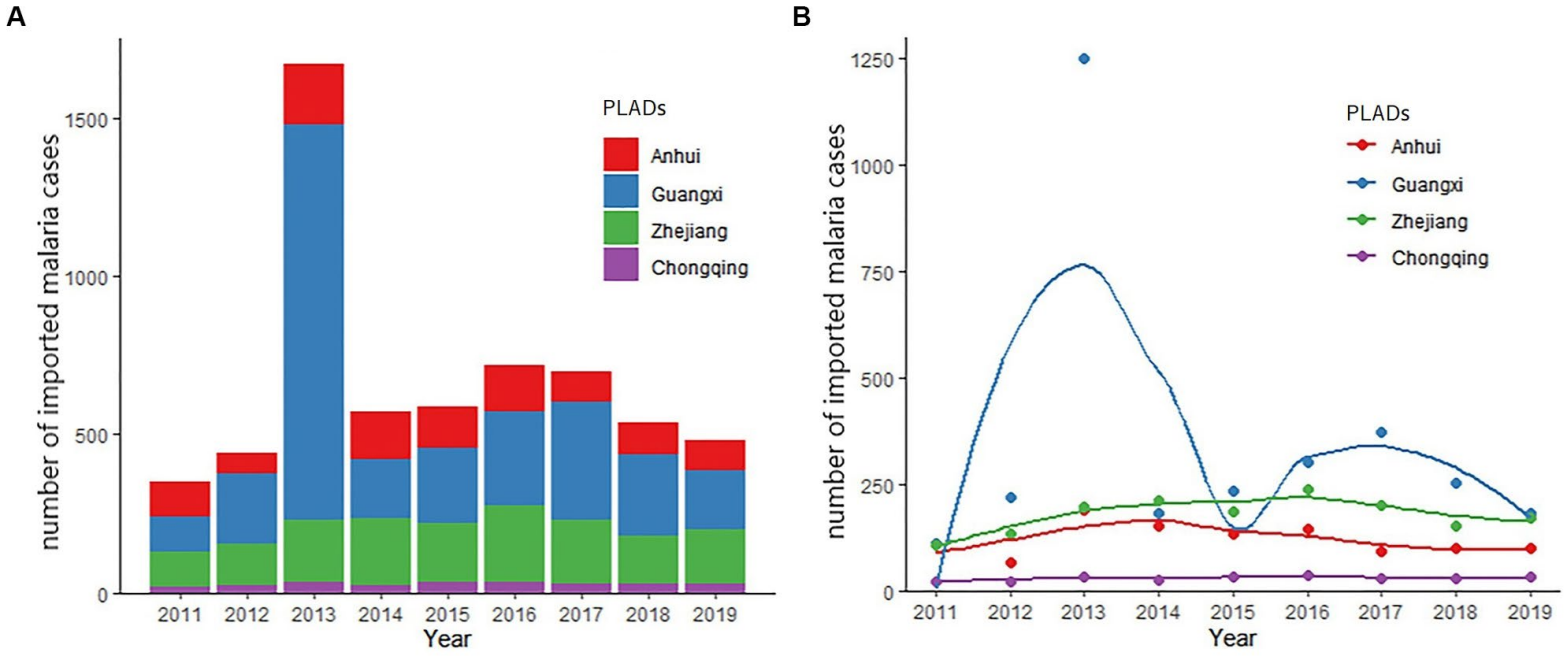
Trends in imported malaria in the four studied PLADs, 2011–2019. **(A)** Number of the total imported malaria cases from 2011 to 2019. **(B)** Trends of imported malaria in the studied PLADs. The smooth curves were created using kernel density estimation to describe the temporal changes in the numbers of malaria cases over time.

As shown in Figure 1, from 2011 to 2019, the annual number of cases of imported malaria was relatively stable, except in 2013. In that year, the confirmed cases of malaria peaked at 1670, because there was a dramatic increase in patient numbers in Guangxi (Figure 1B). In contrast, the numbers of cases ranged from 350 to 721 in all other years studied. The other three provincial areas hold a relatively stable trend of imported malaria cases however variance can also be discovered (Figure 1B). The numbers of cases ranged from 66 (in year 2012) to 191 (in year 2013) in Anhui, 22 (in year 2011) to 36 (in year 2016) in Chongqing, and 110 (in year 2011) to 238 (in year 2016) in Zhejiang.

The peak of imported malaria cases was detected in June (Supplementary Figure S2), and the *Plasmodium* species distribution in the malaria patients differed slightly by month. *P. falciparum* malaria seemed to peak in June, *P. vivax* between April and September, whereas *P. ovale* and *P. malariae* had a relatively stable pattern (Supplementary Figure S3).

### 3.3. Countries and regions of origin

**Figure 2:**
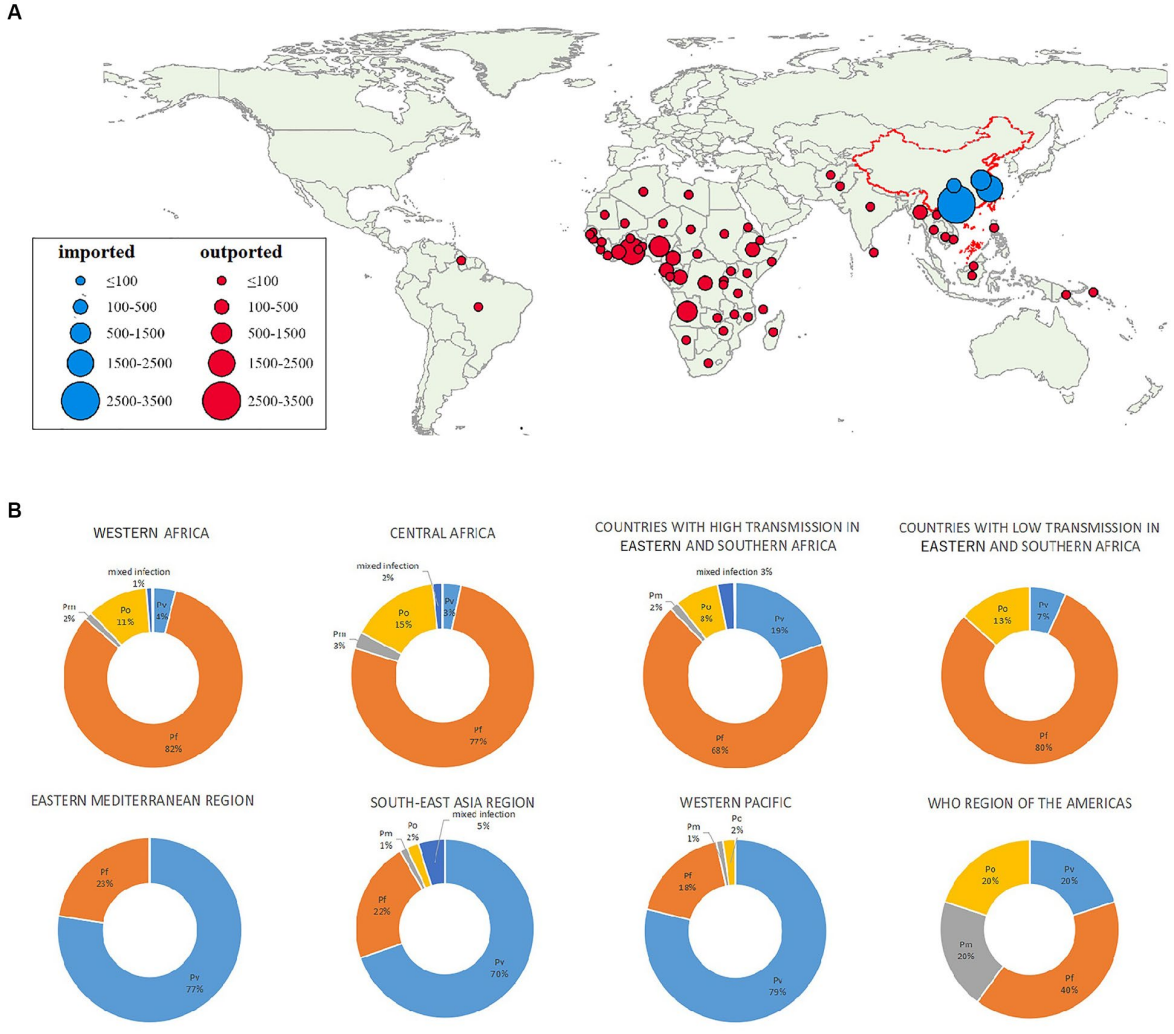
Geographic distribution and species of malarial infections by country and subregion. **(A)** Origins of malaria cases by country. **(B)** Percentages of infections caused by each species of *Plasmodium* by region. *Pf*, *P. falciparum*; *Pv, P. vivax*; *Pm, P. malariae*; *Po, P. ovale*.

The 10 most frequently reported countries of origin of imported malaria accounted for 78.2% (4,740/6,064) of the total cases. Among these countries, four were in Western Africa (Ghana, Nigeria, Angola, and Côte d’Ivoire), with 3,322 (54.8%) reported cases and four were in Central Africa (Cameroon, Democratic Republic of the Congo, Republic of the Congo, and Republic of Equatorial Guinea), with 1,173 (19.3%) reported cases. Myanmar from Southeast Asia (140, 2.3%) and Ethiopia from Eastern Africa (105, 1.6%) were the other two countries rounding up the top 10 origins of imported malaria cases to the area under study in China. Among individual countries, Ghana was the source of most infections, accounting for 34.5% (2095/6064) of the total cases (Figure 2 and Supplementary Figure S4).

According to the WHO Malaria Report 2021, the source countries could be classified into eight regions (Table 1). Specifically, 3,298 (54.4%) cases in this study were imported from the West African Region, followed by Central Africa (1,888, 31.1%) and countries with high transmission rates in Eastern and Southern Africa (458, 7.6%). Cases from South-East Asia accounted for 3.8% (228/6,064) of the total imported cases.

**Table 1:** Source regions of imported malaria by species in the four studied PLADs, 2011–2019.

Malaria species	Central Africa	Countries with high transmission in east and southern Africa	Countries with low transmission in east and southern Africa	Eastern Mediterranean	South-east Asia	West Africa	Western Pacific	Region of the Americas
*P. vivax*	60 (10.5)	89 (15.5)	1 (0.2)	55 (9.6)	159 (27.7)	134 (23.3)	71 (12.4)	1 (0.2)
*P. falciparum*	1,447 (31.6)	313 (6.8)	12 (0.3)	16 (0.3)	50 (1.1)	2,714 (59.3)	16 (0.3)	2 (0)
*P. malariae*	54 (47.8)	7 (6.2)	0 (0)	0 (0)	3 (2.7)	47 (41.6)	1 (0.9)	1 (0.9)
*P. ovale*	293 (41.7)	35 (5)	2 (0.3)	0 (0)	5 (0.7)	363 (51.6)	2 (0.3)	1 (0.1)
Mixed infection	32 (36.4)	13 (14.8)	0 (0)	0 (0)	11 (12.5)	32 (36.4)	0 (0)	0 (0)
NA^*^	2 (18.2)	1 (9.1)	0 (0)	0 (0)	0 (0)	8 (72.7)	0 (0)	0 (0)
Total	1888 (31.1)	458 (7.6)	15 (0.2)	71 (1.2)	228 (3.8)	3,298 (54.4)	90 (1.5)	5 (0.1)

^*^NA: Not available. Origin countries were classified into eight regions according to WHO malaria report of 2021. Eleven cases were not included due to their unavailable origin country. Central Africa: Angola, Burundi, Cameroon, Central African Republic, Chad, Congo, Equatorial Guinea, Gabon, Zaire. West Africa: Algeria, Benin, Burkina Faso, Republic of the Gambia, Ghana, Guinea, Guinea-Bissau, Ivory Coast, Liberia, Mali, Mauritania, Niger, Nigeria, Senegal, Sierra Leone, Togo. Countries with high transmission in East and Southern Africa: Ethiopia, Kenya, Madagascar, Malawi, Mozambique, Rwanda, United Republic of Tanzania, Uganda, Zambia, Zimbabwe. Countries with low transmission in East and Southern Africa: Comoros, Eritrea, Namibia, South Africa. Eastern Mediterranean: Afghanistan, Djibouti, Libya, Pakistan, Somalia, Sudan. WHO Region of the Americas: Brazil, Guyana. Western Pacific: Cambodia, Laos, Malaysia, Papua New Guinea, Philippines, Solomon Islands, Vietnam. Southeast Asia: India, Indonesia, Myanmar (Burma), Sri Lanka, Thailand.

### 3.4. Species of malaria parasites

Out of 6,064 cases recruited during the study period, the etiological agents of 11 cases were not identified at the species level. Among the remaining 6,053 cases, *P. falciparum* was most frequently reported (4,575, 75.6%), followed by *P. ovale* (703, 11.6%), *P. vivax* (574, 9.5%), *P. malariae* (103, 1.7%), and mixed infections (88, 1.5%). An increasing trend in the proportion of *P. ovale* (*β* = 0.03, *p* = 0.003) and a downward trend in the proportion of *P. vivax* (*β* = −0.02, *p* = 0.007) were detected with simple linear regression (Supplementary Figure S5).

Most *P. falciparum* infections (2,714, 59.3%) were imported from Western Africa, followed by Central Africa (1,447, 31.6%). Most *P. vivax* cases reported were acquired in South-East Asia (159/574, 27.7%), Western Africa (134/574, 23.3%), or countries with high transmission rates in Eastern and Southern Africa (89/574, 15.5%). Most *P. ovale* infections were acquired in Western Africa (363/703, 51.6%) and Central Africa (293/703, 41.7%). Similarly, 41.6% (47/113) of the *P. malariae* infections were acquired in Western Africa and 47.8% (54/113) in Central Africa (Table 1 and Figure 2).

### 3.5. Age distribution, sex ratio, and main purposes for traveling

Ages of the patients with imported malaria ranged from 0 to 71 years, of whom the median age of was 40 years (interquartile range, *IQR*: 31–47). Most patients (97.5%) were 20–59 years old (Table 2). The median age of female patients (35 years, *IQR*: 28–45) was generally lower than that of male patients (42 years, *IQ*R: 31–47) in each year studied, except 2013 (Supplementary Figure S6B). Age of imported cases increased significantly by simple linear regression (*β* = 0.583, *p* < 0.001), and the median age increased by 5 years in that period, from 37 years in 2011 to 42 years in 2019 (Table 2).

**Table 2:** Demographic characteristics of malaria patients in the four studied PLADs, 2011–2019 (*n*/proportion or %).

Variable	Categories	2011	2012	2013	2014	2015	2016	2017	2018	2019	Total
Gender (*n*, %)	Male	329 (94)	423 (95.7)	1,635 (97.8)	545 (95.3)	558 (94.9)	684 (94.9)	665 (95.4)	507 (94.1)	457 (94.4)	5,803 (95.7)
	Female	21 (6.0)	19 (4.3)	36 (2.2)	27 (4.2)	30 (5.1)	37 (5.1)	32 (4.6)	32 (5.9)	27 (5.6)	261 (4.3)
Male to female sex ratio (MFSR)		15.7	22.3	45.4	20.2	18.6	18.5	20.8	15.8	16.9	22.2
Age (*n*, %)	≤20	4 (1.1)	12 (2.7)	15 (0.9)	3 (0.5)	3 (0.5)	4 (0.6)	3 (0.4)	6 (1.1)	3 (0.6)	53 (0.9)
	20–29	95 (27.1)	84 (19.0)	359 (21.5)	113 (19.8)	120 (20.4)	127 (17.6)	99 (14.2)	86 (16.0)	68 (14.0)	1,151 (19.0)
	30–39	115 (32.9)	144 (32.6)	513 (30.7)	163 (28.5)	169 (28.7)	203 (28.2)	199 (28.6)	162 (30.1)	131 (27.1)	1799 (29.7)
	40–49	106 (30.3)	152 (34.4)	603 (36.1)	209 (36.5)	224 (38.1)	254 (35.2)	249 (35.7)	163 (30.2)	155 (32.0)	2,115 (34.9)
	50–59	24 (6.9)	46 (10.4)	168 (10.1)	71 (12.4)	65 (11.1)	122 (16.9)	132 (18.9)	106 (19.7)	112 (23.1)	846 (14)
	≥60	6 (1.7)	4 (0.9)	13 (0.8)	13 (2.3)	7 (1.2)	11 (1.5)	15 (2.2)	16 (3.0)	15 (3.1)	100 (1.6)
Purpose of travelling overseas (*n*, %)	Labor export	239 (68.3)	351 (79.4)	1,556 (93.1)	450 (78.7)	453 (77)	546 (75.7)	553 (79.3)	409 (75.9)	357 (73.8)	4,914 (81.0)
	Business trip	42 (12.0)	10 (2.3)	70 (4.2)	76 (13.3)	79 (13.4)	99 (13.7)	109 (15.6)	84 (15.6)	80 (16.5)	649 (10.7)
	Tourism	1 (0.3)	1 (0.2)	2 (0.1)	0 (0)	4 (0.7)	4 (0.6)	4 (0.6)	3 (0.6)	3 (0.6)	22 (0.4)
	Study	3 (0.9)	1 (0.2)	0 (0)	0 (0)	1 (0.2)	3 (0.4)	2 (0.3)	0 (0)	1 (0.2)	11 (0.2)
	Others	65 (18.6)	79 (17.9)	43 (2.6)	46 (8.0)	51 (8.7)	69 (9.6)	29 (4.2)	43 (8.0)	43 (8.9)	468 (7.7)
Total		350	442	1,671	572	588	721	697	539	484	6,064

Males accounted for 95.7% of all the patients with imported malaria. The male to female sex ratio (MFSR) was 17.93. Among all the age groups, this ratio was highest in patients aged ≥60 years (MFSR: 34.8) and in those aged 50–54 years (MFSR: 31.4) (Supplementary Figure S6A). The ratio peaked in 2013, when a large number of gold miners returned to Shang-ling, Guangxi.

The main purposes for traveling abroad were labor export (4,914/6,064, 81.0%) and business trips (649, 10.7%). Other reasons included tourism (22, 0.4%), study (11, 0.2%), and other (468, 7.7%) (Table 2). The proportion of business trips tended to increase in recent years.

### 3.6. First medical visit

Among all the cases of imported malaria, the accurate diagnosis rate at first visit was 74.9% (4,540/6064) between 2011 and 2019. Most patients (2,008/6,064, 33.1%) chose county-level medical institutions for their first medical visit when symptoms developed (Table 3). However, Centers for Disease Control and Prevention (CDCs)/Entry–exit quarantine facilities (EQFs) presented the highest accurate diagnosis rate on the first visit (1,578/1609, 98.1%), followed by provincial-level (466/534, 87.3%), county-level medical institutions (1,734/2,008, 86.4%) and municipal medical institutions (579/716, 80.9%). Town-level medical institutions (159/481, 33.1%) and village-level clinics/private clinics (20/254, 7.9%) had relatively low accurate diagnosis rates at the first visit (Table 3).

**Table 3:** Medical institutions first visited by patients with imported malaria, and the rates of accurate first diagnosis in four PLADs, 2011–2019 (*n*/proportion or %).

**Year**	CDCs/entry-exit quarantines	Provincial medical institutions	Municipal medical institutions	County-level medical institutions	Town-Level medical institutions	Village clinics/private clinics	Others	Total
2011	11 (3.1)	59 (16.9)	23 (6.6)	14 (4)	13 (3.7)	5 (1.4)	225 (64.3)	350
Accurate diagnosis at first visit (%)	11 (100)	59 (100)	22 (95.7)	12 (85.7)	7 (53.8)	1 (20)	0 (0)	112 (32)
2012	39 (8.8)	32 (7.2)	33 (7.5)	70 (15.8)	27 (6.1)	11 (2.5)	230 (52)	442
Accurate diagnosis at first visit (%)	39 (100)	30 (93.8)	24 (72.7)	49 (70)	3 (11.1)	0 (0)	3 (1.3)	148 (33.5)
2013	859 (51.4)	84 (5)	108 (6.5)	450 (26.9)	117 (7)	52 (3.1)	1 (0.1)	1,671
Accurate diagnosis at first visit (%)	849 (98.8)	68 (81)	82 (75.9)	413 (91.8)	51 (43.6)	6 (11.5)	0 (0)	1,469 (87.9)
2014	151 (26.4)	65 (11.4)	84 (14.7)	187 (32.7)	56 (9.8)	27 (4.7)	2 (0.3)	572
Accurate diagnosis at first visit (%)	147 (97.4)	56 (86.2)	69 (82.1)	146 (78.1)	17 (30.4)	0 (0)	1 (50)	436 (76.2)
2015	136 (23.1)	62 (10.5)	89 (15.1)	208 (35.4)	63 (10.7)	29 (4.9)	1 (0.2)	588
Accurate diagnosis at first visit (%)	133 (97.8)	57 (91.9)	74 (83.1)	183 (88)	22 (34.9)	3 (10.3)	0 (0)	472 (80.3)
2016	130 (18)	79 (11)	114 (15.8)	305 (42.3)	55 (7.6)	38 (5.3)	0 (0)	721
Accurate diagnosis at first visit (%)	124 (95.4)	71 (89.9)	94 (82.5)	267 (87.5)	13 (23.6)	2 (5.3)	0 (0)	571 (79.2)
2017	127 (18.2)	46 (6.6)	98 (14.1)	326 (46.8)	73 (10.5)	27 (3.9)	0 (0)	697
Accurate diagnosis at first visit (%)	125 (98.4)	39 (84.8)	80 (81.6)	283 (86.8)	24 (32.9)	1 (3.7)	0 (0)	552 (79.2)
2018	92 (17.1)	57 (10.6)	77 (14.3)	238 (44.2)	39 (7.2)	34 (6.3)	2 (0.4)	539
Accurate diagnosis at first visit (%)	86 (93.5)	47 (82.5)	59 (76.6)	205 (86.1)	12 (30.8)	4 (11.8)	0 (0)	413 (76.6)
2019	64 (13.2)	50 (10.3)	90 (18.6)	210 (43.4)	38 (7.9)	31 (6.4)	1 (0.2)	484
Accurate diagnosis at first visit (%)	64 (100)	39 (78)	75 (83.3)	176 (83.8)	10 (26.3)	3 (9.7)	0 (0)	367 (75.8)
Total	1,609 (26.5)	534 (8.8)	716 (11.8)	2008 (33.1)	481 (7.9)	254 (4.2)	462 (7.6)	6,064
Accurate diagnosis at first visit (%)	1,578 (98.1)	466 (87.3)	579 (80.9)	1734 (86.4)	159 (33.1)	20 (7.9)	4 (0.9)	4,540 (74.9)

### 3.7. Days from visit to diagnosis

Most cases of imported malaria were diagnosed at CDCs/EQFs (2,235/6,064, 36.9%) and county-level medical institutions (2,001/6,064, 33.0%). The percentage of malaria patients diagnosed at CDCs/EQFs declined, whereas those diagnosed at county-level medical institutions increased (Supplementary Table S1).

Among all 6,064 reported cases, most of the reported cases were diagnosed in 1 ~ 3 days by medical institutions (2182/3993, 54.6%) or CDCs/EQFs (1147/1609, 71.3%), respectively. A quarter of them were diagnosed in 1 day (23.9% by medical institutions and 26.3% by CDCs/EQFs) (Supplementary Table S2).

## 4. Discussion

China was declared malaria-free on June 30, 2021 (6). This remarkable achievement was the result of a dedicated effort to prevent malaria over many years, and has become an important monument of malaria elimination in the Asia–Pacific region. However, imported malaria will still pose a threat to China’s malaria-free status until global malaria eradication is finally achieved. There are still malaria vectors widely spread in China. *An. sinensis*, the most widely spread malaria vector in China which is, distributes all over the country. *An. lesteri*, *Anopheles minimus s.l.* and *An. dirus s.l.* are the three other major malaria vectors, sparsely distribute in limited mountainous forest areas, mainly in Southern China (3). In 2011, 20 indigenous cases of malaria were reported in Greece, although it was officially announced malaria-free in 1974 (7, 8). Similarly, a history of malaria reintroduction has been reported in Mauritius (9), Armenia (10), and Sri Lanka (11). Although the reintroduction of malaria into these countries is partly attributable to internal conflicts, migrant workers, visitors, and refugees, the failure to establish prevention of re-establishment (POR) strategies or their poor implementation has had deadly consequences (12). Therefore, in this exploratory epidemiological analysis, we have comprehensively examined the cases of malaria imported into four different PLADs of China between 2011 and 2020, their epidemiological characteristics, and their changing patterns, to offer important information on POR and to protect this momentous achievement.

The cases of imported malaria in three of four provincial areas were quite stable in the years studied, except 2013, when a large number of Chinese gold prospectors were expelled by the Ghana government and returned to Shanglin city in Guangxi Zhuang Autonomous Region within a short period (13). Since 2006, >10,000 inhabitants have traveled abroad from Shanglin for gold mining, most of them to Ghana. This event demonstrates that local governments must be prepared for such unusual, dramatic increases in imported malaria, including provisions for patient diagnosis, epidemiological investigations, treatment, and mosquito surveillance, because the timely detection and treatment of new infections is critical in preventing the re-establishment of local transmission.

In this study, malaria infections were most frequently seen in oversea laborers, who have travelled, lived and worked in malaria endemic countries or territories. Different from European countries where malaria patients are usually travelers and migrants (14), these people have travelled for a longer period of time, also the demographic characteristic and occupation could result in additional risk. According to published studies, most of these laborers are farmers, who are generally poorly educated and unaware of the risk of malaria and personal protection measure to avoid mosquito bites (15). Health education and personal protection equipment are recommended for laborers, especially at the primary health care level. We have also observed an increasing trend of median age of imported malaria. Because older travelers are at higher risk of malaria-related morbidity and mortality than younger people (16), attention should be paid on changes in the age distribution of imported malaria cases. Greater awareness of mosquito protection measures and chemoprophylaxis, and health education before traveling are required.

Similar to other epidemiological studies in other parts of China (17, 18), a weak seasonal peak in June was observed. A possible explanation is that the busy agricultural work during May–September in China requires migrant workers returning to their hometown (19, 20). However, other studies have identified no specific seasonality in imported malaria, so this issue warrants further research.

Sub-Saharan Africa was the main source region for imported malaria in China. Patients were most frequently infected with *P. falciparum*, especially in sub-Saharan African regions. With the high prevalence and relatively high virulence of *P. falciparum*, the importance of its timely diagnosis and effective treatment should not be neglected. Intravenous artesunate for severe malaria and oral artemisinin-based combination therapies for uncomplicated malaria are the first-line treatments for all disease caused by *Plasmodium* species in humans (21). However, evidence of resistance to artemisinin is not only found in the Greater Mekong Subregion, but also been found in sub-Saharan African countries, such as Rwanda (22) and Uganda (23). Though both were Eastern sub-Sahara African countries, the artemisinin resistance still posed a great challenge in the treatment and control of imported *P. falciparum,* and thus, not only for those returning from GMS but also for those returning from African countries, continuous molecular surveillance of *pfk13* mutation in *P. falciparum* are necessary.

Two observations in the Africa Region are noteworthy. About 4% of malaria cases imported from WHO West Africa region involved *P. vivax*, which is inconsistent with the consensus that *P. vivax* infections are uncommon in Western Africa because of the prevalence of the Duffy-negative genetic status. We also noted that during the study period, the number cases of imported *P. ovale* malaria exceeded the number of *P. vivax* infections reported in the African Region, and increased significantly from 2011 to 2019. Our observations are supported by growing evidence suggesting that a Duffy-negative status is no longer a barrier to *P. vivax* infection (24, 25). Our data suggest that *P. vivax* and *P. ovale* infections are underestimated in Africa because these species are not routinely identified in most countries. Besides, though we did not detect any case infected with *Plasmodium knowlesi* (*P. knowlesi*) in the four studied areas from 2011 to 2019, the expanding prevalence of *P. knowlesi* infection in Southeast Asia should never be ignored for the control and elimination of malaria. *P. knowlesi* infection occurs in forested areas where monkeys and humans coexist (26). The majority of *P. knowlesi* malaria infections cause mild clinical manifestations, with an estimated 6%–9% of severe cases and increasingly frequently reported asymptomatic infections in the last decade (27). PCR methods are important to confirm *P. knowlesi* infection, since the morphology of rings of *P. knowlesi* in thin blood film smears is similar to *P. falciparum*, as well as trophozoites and schizozoites similar with *P. malariae.* In China, a network of reference laboratories, including a national laboratory and provincial laboratories in CDCs, have been built since 2013. PCR is used in reference laboratories to identify and double check the species responsible for malaria infections. However, the large number of imported *P. vivax* and *P. ovale* cases is also a challenge in China, because their less-typical symptoms or even asymptomatic presentation require effective diagnostic tools and sensitive monitoring systems, not only in CDCs but also in medical institutions.

In this study, we found that most malaria patients first visited county-level institutions when seeking medical help in China, and that nearly a third of imported infections were diagnosed and treated at county-level institutions. However, the rate of correct diagnosis at county-level institutions was lower than that at CDCs/EQFs and provincial-level institutions. Our study also showed that CDCs/EQFs had a quicker response to patients with malaria than medical institutions, insofar as more patients were diagnosed within 3 days of visiting a CDCs/EQFs than within 3 days of visiting a medical institution. Based on our data, medical institutions, especially county-level institutions, should be the primary targets of measures to improve the sensitivity of the surveillance system in China.

This study had several limitations. First, no information on the severity or mortality of the malaria reported was included in our analysis. Second, the number of travelers returning to China from various countries was not obtained, so we could not estimate and compare the incidence of malaria imported by the country of origin. Third, the specific origins of malaria within the source countries were not investigated, which limited further interpretation of the data.

## 5. Conclusion

In this study, we thoroughly analyzed the surveillance data on malaria imported into four PLADs of China between 2011 and 2019. We detected a quite stable trend in imported malaria cases, suggesting that imported malaria still poses a threat to the malaria-free status of China. Our analysis also demonstrated that county-level institutions should be the primary targets of measures to improve the sensitivity of surveillance systems in China and to prevent the re-establishment of malaria. We also observed an increasing trend in *P. ovale* and the persistence of *P. vivax* infections in Western Africa throughout these years, indicating that the prevalence of *P. ovale* and *P. vivax* is underestimated in Western Africa. Efficient diagnostic tools and sensitive monitoring systems are required to identify the *Plasmodium* species prevalent in Africa.

## Data availability statement

The data analyzed in this study is subject to the following licenses/restrictions: The datasets presented in this article are not readily available because the ISPDCP is not a publicly available data repository. Requests to access these datasets should be directed to DW, wangdq@nipd.chinacdc.cn.

## Author contributions

DW and WR conceived the study. XW, WX, FL, KL, and TZ designed and carried out the analysis. XZ, FL, and JZ prepared the datasets. LY, SA, DW, and WR advised on the analysis. XW and WX wrote the manuscript. XW, WX, FL, KL, TZ, LY, XZ, JZ, SA, DW, and WR contributed to the interpretation of results. All authors contributed to the article and approved the submitted version.

## Funding

This work was funded by grants from the Major Health Science and Technology Projects in Zhejiang Province (Grant No. WKJ-ZJ-2119) and Medical Research Program of Zhejiang Province (Grant No. 2020PY038 and 2022KY723).

## Conflict of interest

The authors declare that the research was conducted in the absence of any commercial or financial relationships that could be construed as a potential conflict of interest.

## Publisher’s note

All claims expressed in this article are solely those of the authors and do not necessarily represent those of their affiliated organizations, or those of the publisher, the editors and the reviewers. Any product that may be evaluated in this article, or claim that may be made by its manufacturer, is not guaranteed or endorsed by the publisher.

## Footnotes

^1^ http://cran.r-project.org
