# Supplementary material for: Increasing incidence of Plasmodium ovale and persistent reporting of Plasmodium vivax in imported malaria cases: an analysis of 9-year surveillance data in four areas of China
Source: Front Public Health. 2023 Jun 28;11:1203095. doi: 10.3389/fpubh.2023.1203095 (PMC10338171; doi:10.3389/fpubh.2023.1203095)
Supplement: Supplementary file 1 [file Data_Sheet_1.docx]

Supplementary Material

Increasing Plasmodium ovale and persistence of Plasmodium vivax in imported malaria: an analysis of 9-year surveillance data in four provinces of China

**Xiaoxiao Wang1,+, Wenjie Xu1,+, Fei Luo2, Kangming Lin3, Tao Zhang4, Linong Yao1, Xuan Zhang1, Jiaqi Zhang1,** **Sarah Auburn5,6, Duoquan Wang7,*, Wei Ruan1,***

**+These authors contributed equally to this work and share first authorship**

*** Correspondence:** Duoquan Wang: [wangdq@nipd.chinacdc.cn](mailto:wangdq@nipd.chinacdc.cn); Wei Ruan: [wruan@cdc.zj.cn](mailto:wruan@cdc.zj.cn)

## 1 Supplementary Figures


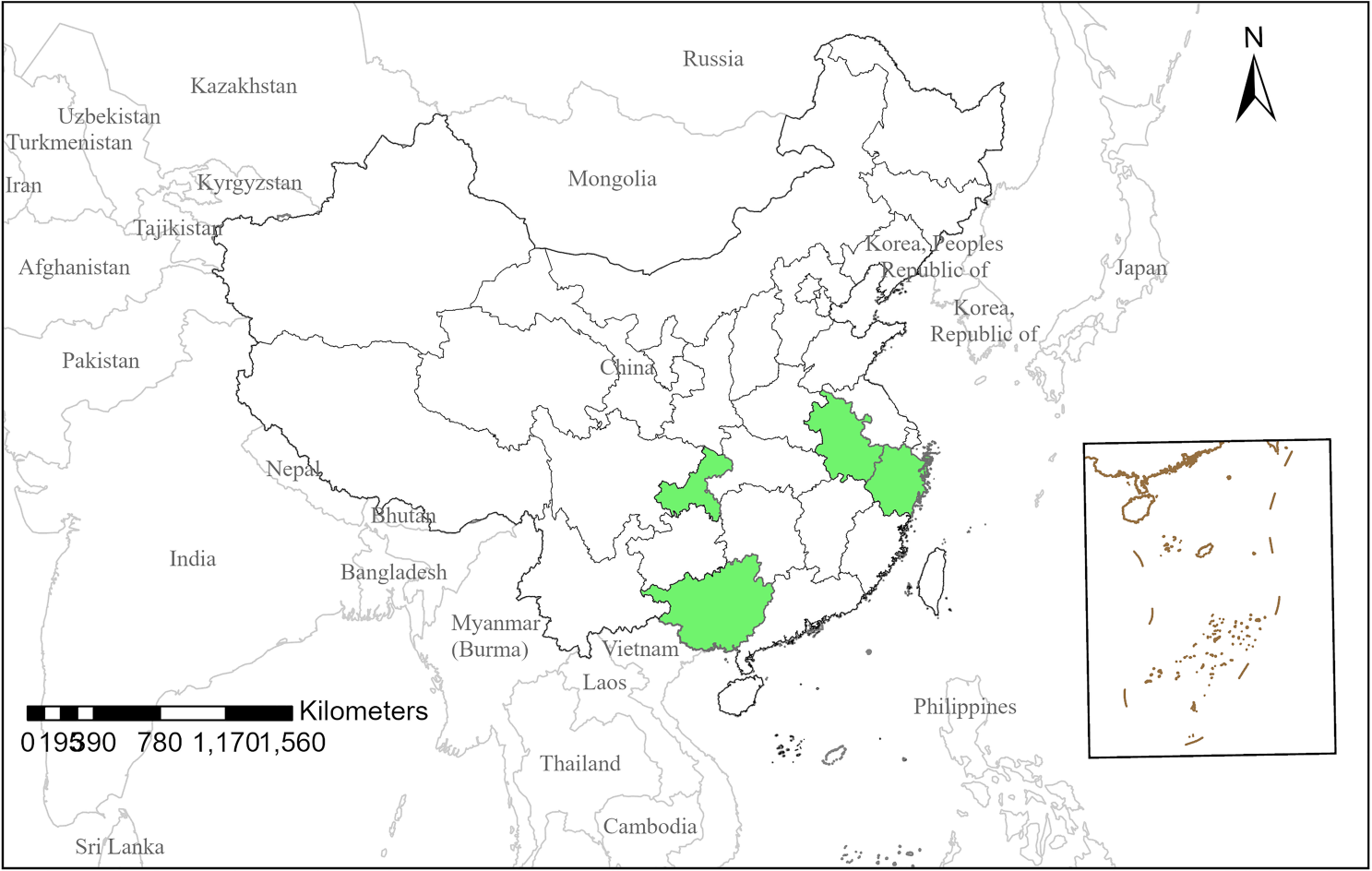


**Supplementary Figure 1.** Spatial distribution of the provinces studied, China.


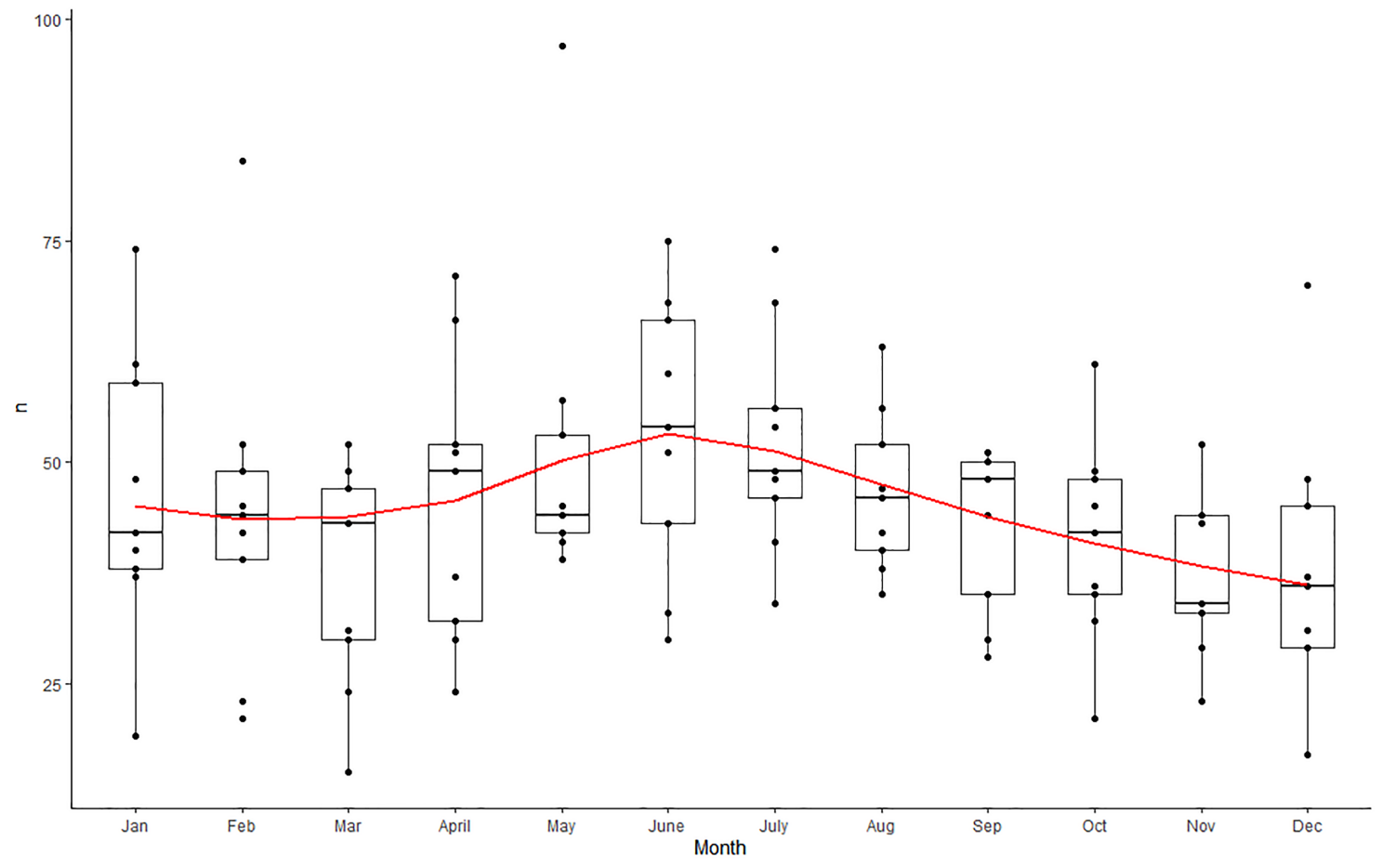


**Supplementary Figure 2.** Seasonal distribution of imported malaria in studied provinces, 2011-2019

**
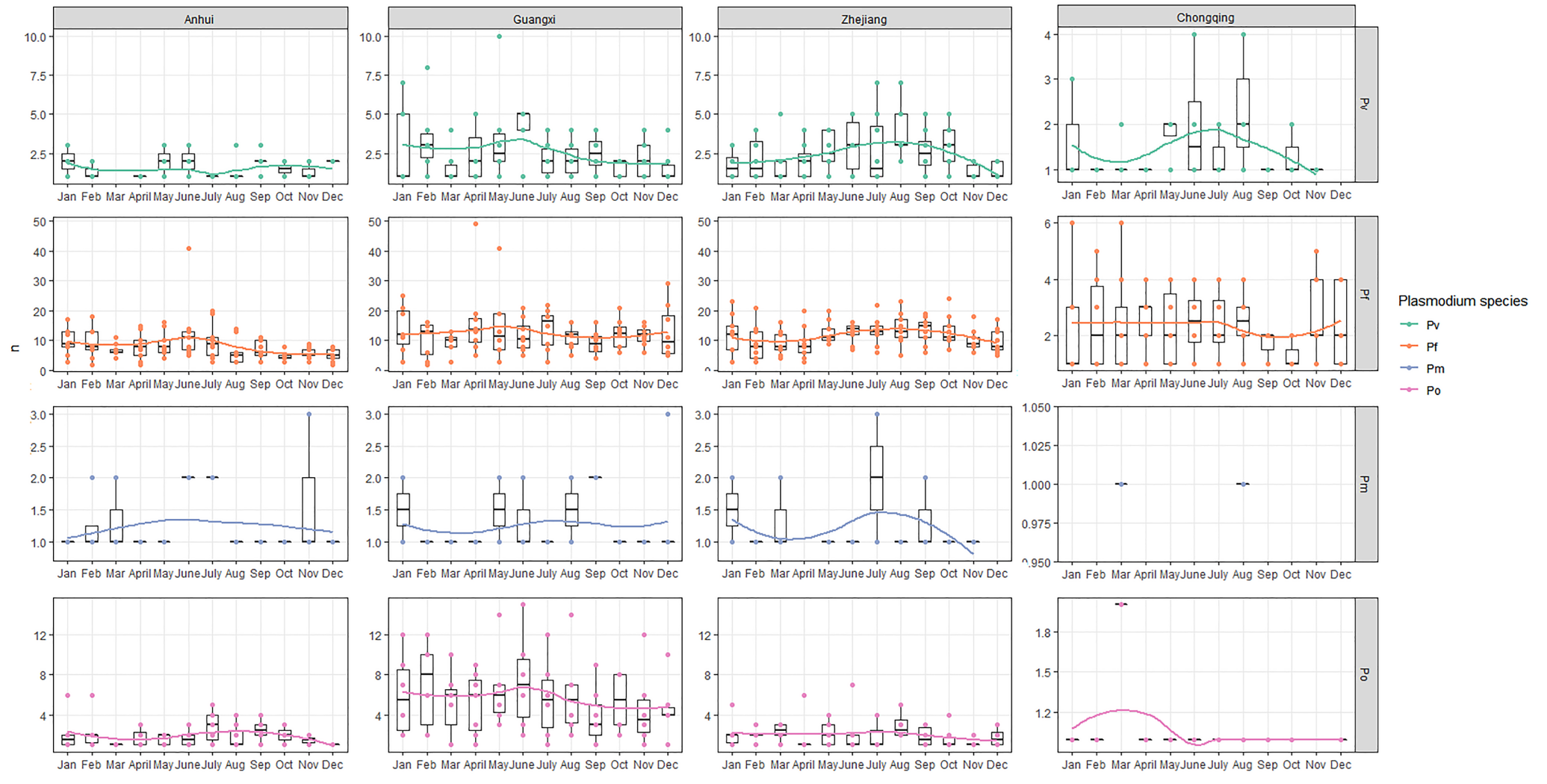
**

**Supplementary Figure 3.** Seasonal patterns of infection by malaria species

**
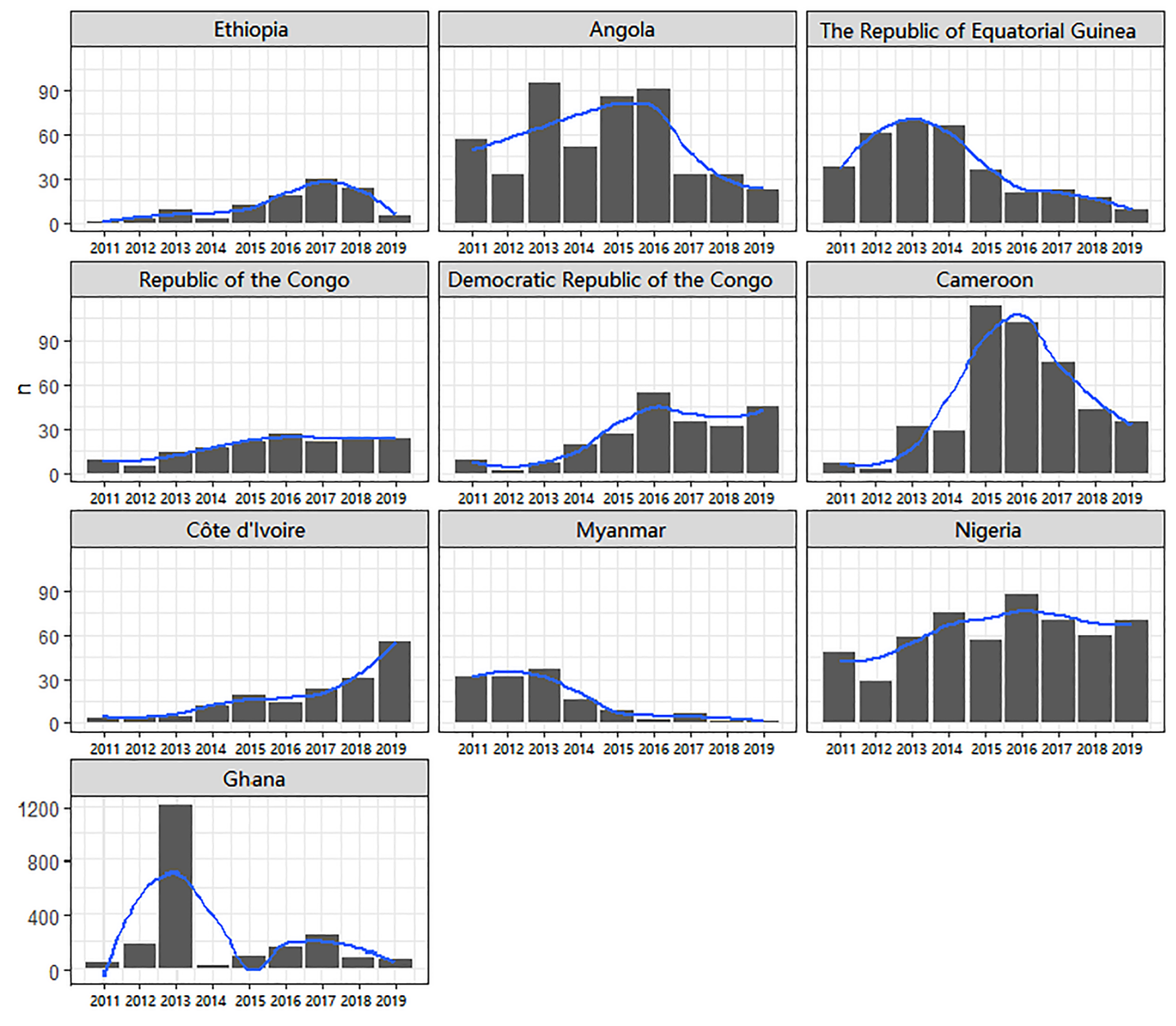
**

**Supplementary Figure 4.** Trend in imported malaria from 10 countries most frequently reported, 2011–2019. Blue lines are loess regression lines fitting smooth curves to the trends for malaria imported into the studied provinces, 2011-2019

**
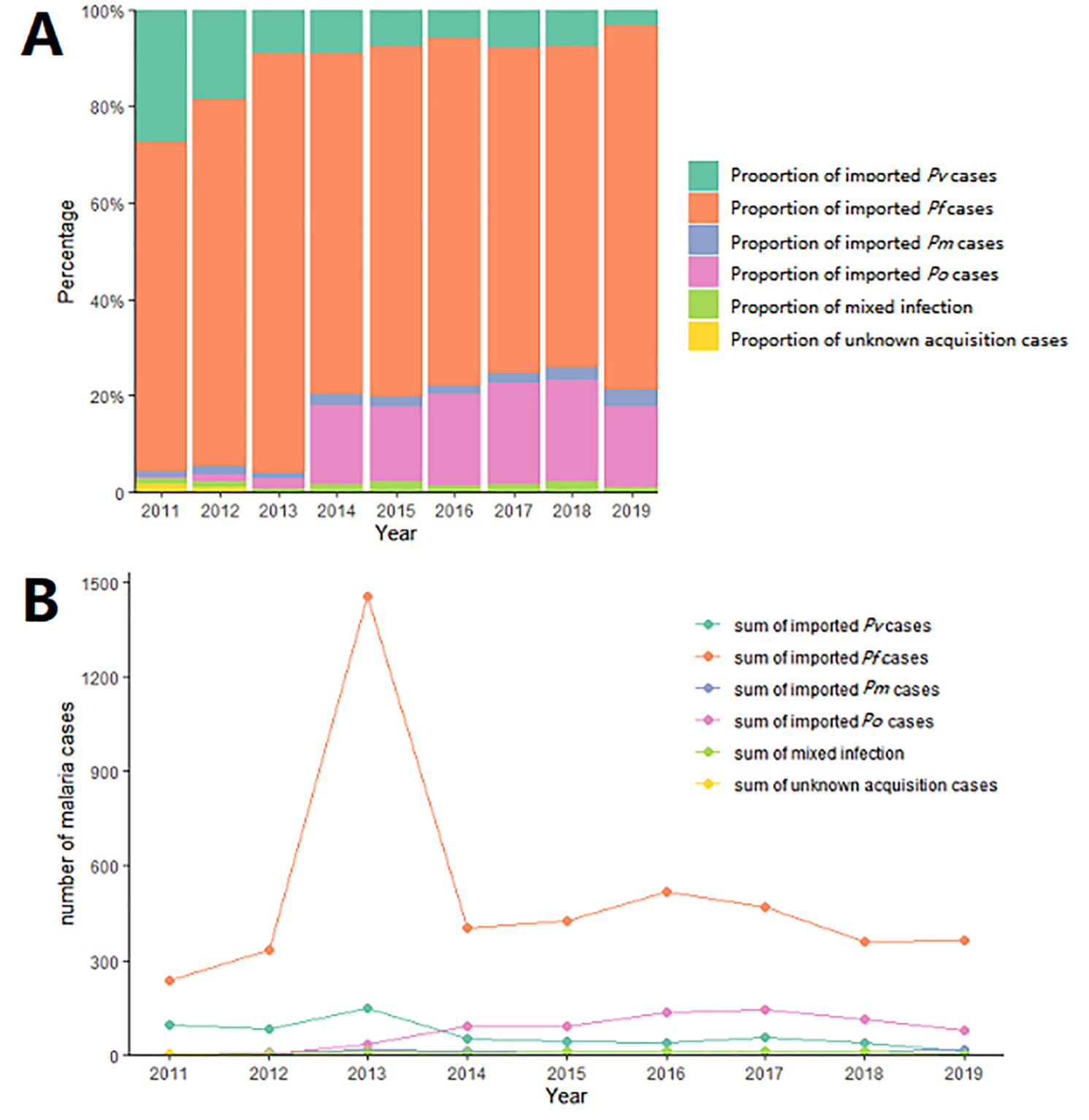
**

**Supplementary Figure 5.** Malarial species imported in 2011–2019, represented by percentiles (A) and line charts (B)

**
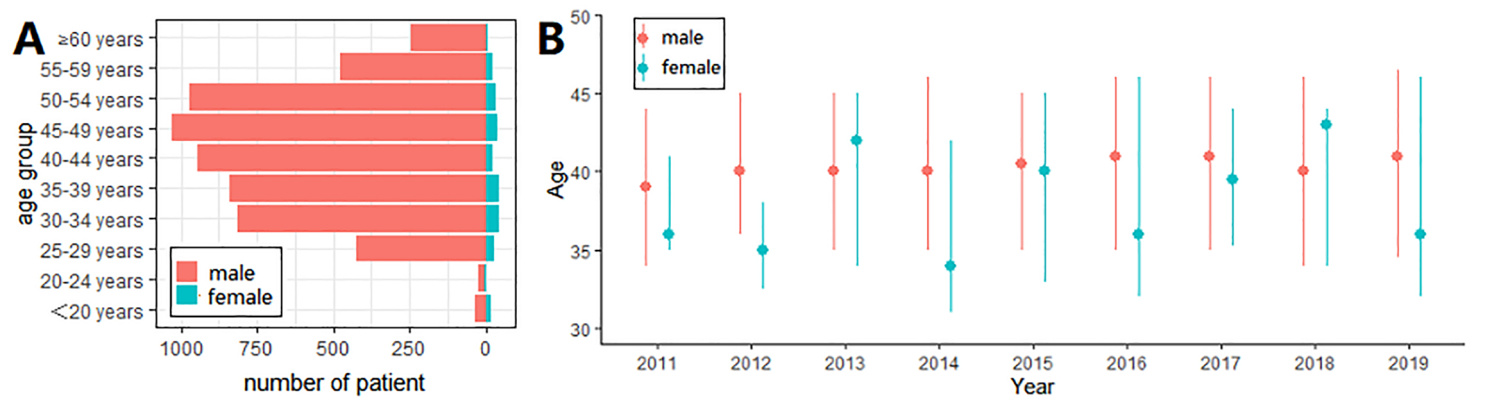
**

**Supplementary Figure 6.** Age distribution and sex ratio of imported malaria in studied provinces, 2011-2019. A. Distribution of age by sex. B. Median age (with error bars indicating interquartile ranges) of both sexes when affected

## 2 Supplementary Tables

**Supplementary Table 1.** Institutions that diagnosed imported malaria cases in four provinces, 2011–2019 (n/proportion or %)

| **Year** | **CDCs/ Entry-exit quarantines** | **Provincial medical institutions** | **Municipal medical institutions** | **County-level medical institutions** | **Town-Level medical institutions** | **Village clinics/private clinics** | **Total** |
| --- | --- | --- | --- | --- | --- | --- | --- |
| 2011 | 104 (29.7) | 109 (31.1) | 83 (23.7) | 44 (12.6) | 10 (2.9) | 0 (0) | 350 |
| 2012 | 301 (68.1) | 59 (13.3) | 38 (8.6) | 44 (10) | 0 (0) | 0 (0) | 442 |
| 2013 | 957 (57.3) | 137 (8.2) | 104 (6.2) | 433 (25.9) | 39 (2.3) | 1 (0.1) | 1671 |
| 2014 | 198 (34.6) | 98 (17.1) | 93 (16.3) | 170 (29.7) | 12 (2.1) | 1 (0.2) | 572 |
| 2015 | 172 (29.3) | 90 (15.3) | 104 (17.7) | 206 (35) | 16 (2.7) | 0 (0) | 588 |
| 2016 | 167 (23.2) | 91 (12.6) | 138 (19.1) | 308 (42.7) | 17 (2.4) | 0 (0) | 721 |
| 2017 | 153 (22) | 48 (6.9) | 146 (20.9) | 331 (47.5) | 19 (2.7) | 0 (0) | 697 |
| 2018 | 112 (20.8) | 60 (11.1) | 117 (21.7) | 240 (44.5) | 10 (1.9) | 0 (0) | 539 |
| 2019 | 71 (14.7) | 63 (13) | 115 (23.8) | 225 (46.5) | 10 (2.1) | 0 (0) | 484 |
| Total | 2235 (36.9) | 755 (12.5) | 938 (15.5) | 2001 (33) | 133 (2.2) | 2 (0) | 6064 |

**Supplementary Table 2.** Days from first visit to diagnosis of imported malaria cases in different institutions in four provinces, 2011–2019 (n/proportion or %)

| **Year** | **n** | **Medical institutions*** | | | | **CDCs/Entry-exit quarantines** | | | |
| --- | --- | --- | --- | --- | --- | --- | --- | --- | --- |
|  |  | **0d** | **1-3d** | **>3d** | **Total** | **0d** | **1-3d** | **>3d** | **Total** |
| 2011 | 125 | 4 (3.5) | 66 (57.9) | 44 (38.6) | 114 | 9 (81.82) | 0 (0) | 2 (18.18) | 11 |
| 2012 | 212 | 0 (0) | 92 (53.2) | 81 (46.8) | 173 | 36 (92.31) | 0 (0) | 3 (7.69) | 39 |
| 2013 | 1670 | 342 (42.2) | 321 (39.6) | 148 (18.2) | 811 | 85 (9.9) | 764 (88.94) | 10 (1.16) | 859 |
| 2014 | 570 | 41 (9.8) | 276 (65.9) | 102 (24.3) | 419 | 71 (47.02) | 72 (47.68) | 8 (5.3) | 151 |
| 2015 | 587 | 104 (23.1) | 260 (57.6) | 87 (19.3) | 451 | 55 (40.44) | 78 (57.35) | 3 (2.21) | 136 |
| 2016 | 721 | 154 (26.1) | 331 (56) | 106 (17.9) | 591 | 66 (50.77) | 60 (46.15) | 4 (3.08) | 130 |
| 2017 | 697 | 193 (33.9) | 275 (48.2) | 102 (17.9) | 570 | 41 (32.28) | 84 (66.14) | 2 (1.57) | 127 |
| 2018 | 537 | 84 (18.9) | 268 (60.2) | 93 (20.9) | 445 | 35 (38.04) | 51 (55.43) | 6 (6.52) | 92 |
| 2019 | 483 | 31 (7.4) | 293 (69.9) | 95 (22.7) | 419 | 26 (40.63) | 38 (59.38) | 0 (0) | 64 |
| Total | 6064 | 953 (23.9) | 2182 (54.6) | 858 (21.5) | 3993 | 424 (26.3) | 1147 (71.3) | 38 (2.4) | 1609 |
| *Medical institutions include provincial, municipal, county-level, and town-Level medical institutions and village clinics or private clinics. | | | | | | | | | |
